# Evaluation of Case Volumes of a Heart Transplant Program and Short-term Outcomes After Changes in the United Network for Organ Sharing Donor Heart Allocation System

**DOI:** 10.1001/jamanetworkopen.2020.17513

**Published:** 2020-09-18

**Authors:** Makoto Mori, Lynn Wilson, Ayyaz Ali, Tariq Ahmad, Muhammad Anwer, Daniel Jacoby, Arnar Geirsson, Harlan M. Krumholz

**Affiliations:** 1 Section of Cardiac Surgery, Yale School of Medicine, New Haven, Connecticut; 2 Center for Outcomes Research and Evaluation, Yale New Haven Hospital, New Haven, Connecticut; 3 Heart and Vascular Center, Yale New Haven Hospital, New Haven, Connecticut; 4 Department of Cardiac Surgery, Hartford Hospital, Hartford, Connecticut; 5 Section of Cardiovascular Medicine, Department of Internal Medicine, Yale School of Medicine, New Haven, Connecticut

## Abstract

**Importance:**

Institution-level strategic changes may be associated with heart transplant volume and outcomes.

**Objective:**

To describe changes in practice that markedly increased heart transplant volume at a single center, as well as associated patient characteristics and outcomes.

**Design, Setting, and Participants:**

A pre-post cohort study was conducted of 107 patients who underwent heart transplant between September 1, 2014, and August 31, 2019, at Yale New Haven Hospital before (September 1, 2014, to August 31, 2018; prechange era) and after (September 1, 2018, to August 31, 2019; postchange era) a strategic change in patient selection by the heart transplant program.

**Exposure:**

Strategic change in donor and recipient selection at Yale New Haven Hospital that occurred in August 2018.

**Main Outcomes and Measures:**

Outcome measures were transplant case volume, donor and recipient characteristics, and 180-day survival.

**Results:**

A total of 49 patients (12.3 per year; 20 women [40.8%]; median age, 57 years [interquartile range {IQR}, 50-63 years]) received heart transplants in the 4 years of the prechange era and 58 patients (58 per year; 19 women [32.8%]; median age, 57 years [IQR, 52-64 years]) received heart transplants in the 1 year of the postchange era. Organ offers were more readily accepted in the postchange era, with an offer acceptance rate of 20.5% (58 of 283) compared with 6.4% (49 of 768) in the prechange era *(P <* .001). In the postchange era, donor hearts were accepted with a higher median number of prior refusals by other centers than in the prechange era (16.5 [IQR, 6-38] vs 3 [IQR, 1-6]; *P* < .001). Hearts accepted in the postchange era were from older donors than in the prechange era (median age, 40 years [IQR, 29-48 years] vs 30 years [IQR, 24-42 years]; *P* < .001). Recipients had a significantly shorter time on the waiting list in the postchange era compared with prechange era (median, 41 days [IQR, 12-289 days] vs 242 days [IQR, 135-428 days]; *P* < .001). More patients were supported on temporary circulatory assist devices preoperatively in the postchange era than the prechange era (14 [24.1%] vs 0; *P* < .001). Survival rates at 180 days were not significantly different (43 [87.8%] in the prechange era vs 52 [89.7%] in the postchange era). Mortality while on the waiting list was similar (2.8 deaths per year in the prechange era vs 3 deaths per year in the postchange era). During the comparable time period, 4 other regional centers had volume change ranging from −10% to 68%, while this center’s volume increased by 374%.

**Conclusions and Relevance:**

This study suggests that strategic changes in donor heart and recipient selection may significantly increase the number of heart transplants while maintaining short-term outcomes comparable with more conservative patient selection. Such an approach may augment the allocation of currently unused donor hearts.

## Key Points

**Question:**

Could a rapid transition from conservative to aggressive selection of donor recipients for heart transplant be achieved safely?

**Findings:**

In this pre-post cohort study that included 49 heart transplants performed in the prechange era and 58 heart transplants performed in the postchange era before and after patient selection strategy changes in an advanced heart failure program, recipients had a significantly shorter time on the waiting list in the postchange era compared with the prechange era. Survival at 180 days was not significantly different between the eras (88% in the prechange era vs 90% in the postchange era).

**Meaning:**

Strategic changes in donor heart and recipient selections may substantially increase the number of heart transplants while maintaining short-term outcomes comparable with more conservative patient selection.

## Teaser

This pre-post cohort study describes changes in practice that markedly increased heart transplant volume at a single center, as well as associated patient characteristics and outcomes.

## Introduction

In heart transplantation, there is uncertainty about who is eligible to receive donor hearts and which hearts are acceptable.^1,2^ For example, donor sequence number dictates how likely it is that the heart will be used, but donor sequence number correlates poorly with posttransplant outcomes.^3^ In addition, donor hearts traditionally perceived as high risk, including those from hepatitis C virus–positive donors^4^ and those with donation after circulatory death status,^5^ are being considered as potentially suitable donor hearts. The evolving perception of acceptable donor hearts may lead centers to apply a more inclusive set of criteria for accepting hearts. However, it remains unknown how such multifaceted expansion for donor heart selection may be associated with transplant case volume and outcomes.

Our hospital (Yale New Haven Hospital) experienced a 5-fold increase in heart transplant volume after restructuring of the heart failure service, change in surgical leadership, and adoption of a more aggressive philosophy on donor heart selection, accepting higher-risk donor hearts that coincided with implementation of the new United Network for Organ Sharing (UNOS) donor heart allocation system in the United States, which was implemented in 2018.^6^ In this study, we investigated the changes in donor and recipient characteristics that occurred during the case volume increase. We also compared outcomes before and after the increase. Our goal is to provide accountability and insight regarding the increase in volume and to extract lessons for other centers contemplating a change in practice.

## Methods

### Patients and Data Sources

We conducted a pre-post cohort study of all patients who underwent heart transplant between September 1, 2014, and August 31, 2019, at Yale New Haven Hospital, comparing before and after the restructuring of the advanced heart failure program with a change in leadership and donor selection philosophy. As changes occurred in mid-August 2018, we dichotomized the cohort into those who underwent surgery before (prechange era) and after (postchange era) September 1, 2018. We used institutional electronic medical records and Scientific Registry of Transplant Recipients data. We used Scientific Registry of Transplant Recipients data for 4 regional centers (Figure) to infer the association of the UNOS allocation system change with volume increase. The Yale Institutional Review Board approved the study and waived individual consent because this observational research presented no more than minimal risk. This study followed the Strengthening the Reporting of Observational Studies in Epidemiology (STROBE) reporting guideline for cohort studies.

**Figure.**
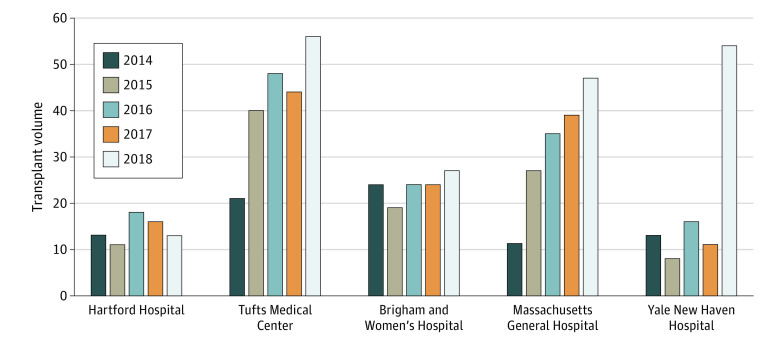
Annual Heart Transplant Volume at Regional Centers Annual transplant case volume at our center and 4 regional centers. Each year represents July 1 of that year to June 30 of the following year.

### Structural and Strategic Changes in the Program

The following strategic changes occurred between the prechange era and the postchange era: (1) change in the surgical directorship in the advanced heart failure service, (2) change in the heart failure leadership’s philosophy to a more patient-centered donor heart use to provide more transplant opportunities, (3) hospital administration’s support to hire a dedicated procurement surgeon and additional transplant coordinator, and (4) increased surgical attending physician involvement in pretransplant listing and weekly multidisciplinary rounding on inpatients awaiting transplant.

During both eras, the transplant surgeon on call screened initial offers of donor hearts. Then, all donors considered for transplant were discussed with the heart failure cardiologist on call. In cases for donor offers meeting the extended criteria in the postchange era, the surgical and medical leadership were also involved in the discussion. Such multidisciplinary discussion was routine in the postchange era. In the postchange era, heart size mismatches were tolerated more aggressively, as were donors of higher age and high-risk donors, who were predominantly donors with prior drug use. We did not accept hepatitis C virus–positive donors during either era.

### Variables and Outcome Measures

We evaluated recipient demographic characteristics, waiting list status, and comorbidity data. Laboratory values were the closest within 30 days and prior to the transplant. Recipient preoperative inotrope use was defined as use of at least 1 inotrope at the time of entering the operating room. For donor hearts, we evaluated high-risk status, number of offers made prior to our center accepting the heart, and donor demographics and comorbidities. We used 180-day survival as the outcome to ensure that all patients completed the follow-up during the period.

### Statistical Analysis

We summarized continuous data with median values with interquartile ranges (IQRs). We used the Wilcoxon rank-sum test to compare continuous variables and the χ^2^ test or Fisher exact test to compare categorical variables. All *P* values were from 2-sided tests and results were deemed statistically significant at *P* < .05. Analysis was performed with SAS, version 9.4 (SAS Institute Inc).

## Results

A total of 49 patients (12.3 per year; 20 women [40.8%]; median age, 57 years [IQR, 50-63 years]) received heart transplants in the 4 years of the prechange era and 58 patients (58 per year; 19 women [32.8%]; median age, 57 years [IQR, 52-64 years]) received heart transplants in the first year of the postchange era (Table 1). The organ offer acceptance rate in the postchange era was 20.5% (58 of 283), compared with 6.4% (49 of 768) in the prechange era *(P <* .001). In the postchange era, donor hearts were accepted with a higher median number of refusals by other centers than in the prechange era (16.5 [IQR, 6-38] vs 3 [IQR, 1-6]; *P* < .001). Hearts accepted in the postchange era were from older donors than in the prechange era (median age, 40 years [IQR, 29-48 years] vs 30 years [IQR, 24-42 years]; *P* < .001); donors in the postchange era also had more comorbidities than donors in the prechange era. Numbers of deaths while on the waiting list were similar between the 2 eras (2.8 deaths per year in the prechange era vs 3 deaths per year in the postchange era). Five patients who were dually listed with another center underwent a transplant at another center in the prechange era while no dually listed patients underwent a transplant at another center in the postchange era. The number of durable left ventricular assist devices implanted was lower in the postchange era (29.5 cases per year in the prechange era vs 12 cases per year in the postchange era) (Table 2). Leading reasons for the refusal of donor offers were similar between the 2 eras, with donor size mismatch comprising 55.6% of refusals (400 of 719) in the prechange era and 58.7% of refusals (132 of 225) in the postchange era, followed by donor age or quality, comprising 22.3% of refusals (160 of 719) in the prechange era and 20.9% of refusals (47 of 225) in the postchange era (Table 3).

**Table 1.** Recipient Characteristics by Transplant Era

Characteristic	Era, No. (%)		*P* value
	Prechange (n = 49)	Postchange (n = 58)	
Waiting list duration, median (IQR), d	242 (135-428)	41 (12-289)	<.001
UNOS status			
Former			
1A	45 (91.8)	4 (6.9)	NA
1B	4 (8.2)	0	
2	0	2 (3.4)	
Current			
1	NA	4 (6.9)	NA
2	NA	14 (24.1)	
3	NA	13 (22.4)	
4	NA	12 (20.7)	
5	NA	2 (3.4)	
6	NA	7 (12.1)	
Age, median (IQR), y	57 (50-63)	57 (52-64)	.53
Female sex	20 (40.8)	19 (32.8)	.74
Use of ECMO before transplant	0	4 (6.9)	.12
Use of durable LVAD before transplant	31 (63.3)	12 (20.7)	<.001
Use of IABP before transplant	0	10 (17.2)	.002
Heart-kidney transplant	1 (2.0)	6 (10.3)	.08
Psychiatric history	7 (14.3)	11 (19.0)	.52
Prior cardiac surgery (including LVAD)	33 (67.3)	24 (41.4)	.007
Stroke	13 (26.5)	13 (22.4)	.25
Preoperative mechanical ventilatory support	0	3 (5.2)	.25
Implanted defibrillator	33 (67.3)	30 (51.7)	.10
Preoperative inotropes	26 (53.1)	26 (44.8)	.40
Dialysis	0	2 (3.4)	.50
Smoking within 1 y of transplant	4 (8.2)	2 (3.4)	.41
Sodium, median (IQR), mEq/L	139 (137-141)	137 (135-141)	.23
Creatinine, median (IQR), mg/dL	1.21 (1.00-1.42)	1.15 (0.88-1.56)	.88
Total bilirubin, median (IQR), mg/dL	0.5 (0.3-0.9)	0.6 (0.4-1.0)	.14
Hematocrit, median (IQR), %	34.9 (33.2-37.7)	35.6 (30.9-40.5)	.69
Platelets, median (IQR), ×10^3^/μL	212 (172-255)	204 (161-256)	.50
180-d Survival	43 (87.8)	52 (89.7)	.81
ICU length of stay, median (IQR)	7 (5-9)	10 (7-17)	<.001

Abbreviations: ECMO, extracorporeal membrane oxygenation; IABP, intra-aortic balloon pump; ICU, intensive care unit; IQR, interquartile range; LVAD, left ventricular assist device; NA, not applicable; UNOS, United Network for Organ Sharing.

SI conversion factors: To convert sodium to millimoles per liter, multiply by 1.0; creatinine to micromoles per liter, multiply by 88.4; bilirubin to micromoles per liter, multiply by 17.104; hematocrit to proportion of 1.0, multiply by 0.01; and platelets to ×10^9^ per liter, multiply by 1.0.

**Table 2.** Donor and Center Characteristics by Transplant Era

Variable	Era, No. (%)		*P* value
	Prechange (n = 49)	Postchange (n = 58)	
Center data			
Offers received, No.	768	283	NA
Offer acceptance rate	49 (6.4)	58 (20.5)	<.001
Refusals by other centers before donor heart accepted by our center, median (IQR), No.	3 (1-6)	16.5 (6-38)	<.001
Death while on waiting list (per year), No.	2.8	3	NA
Dually listed patients who underwent transplant at another center, No.	5	0	NA
LVAD case volume (per year), No.	29.5	12	NA
Destination therapy LVAD (per year), No.	24	8	NA
Donor data			
Donor age, median (IQR), y	30 (24-42)	40 (29-48)	<.001
Female donor	13 (26.5)	12 (20.7)	.48
Blood type O	17 (34.7)	33 (56.9)	.02
High-risk donors	19 (38.8)	26 (44.8)	.48
Inotrope use at procurement	3 (6.1)	4 (6.9)	>.99
Diabetes	2 (4.1)	8 (13.8)	.10
Hypertension	7 (14.3)	20 (34.5)	.01

Abbreviations: IQR, interquartile range; LVAD, left ventricular assist device; NA, not applicable.

**Table 3.** Reasons for Donor Offer Refusals by the Era

Refusal reason	Era, No. (%)	
	Prechange (n = 719)	Postchange (n = 225)
Donor size or weight	400 (55.6)	132 (58.7)
Donor age or quality	160 (22.3)	47 (20.9)
No serum for crossmatching	46 (6.4)	2 (0.9)
Organ-specific donor issue	44 (6.1)	2 (0.9)
Distance to travel or ship the heart is too great	20 (2.8)	10 (4.4)
Patient received transplant or transplant in progress	10 (1.4)	25 (11.1)
Positive crossmatch	9 (1.3)	0
Unacceptable antigens	9 (1.3)	1 (0.4)
Patient ill or unavailable	7 (1.0)	4 (1.8)
Other	14 (1.9)	2 (0.9)

Recipients had a significantly shorter time on the waiting list in the postchange era compared with prechange era (median, 41 days [IQR, 12-289 days] vs 242 days [IQR, 135-428 days]; *P* < .001) (Table 1). Prechange era patients received pretransplant support from durable left ventricular assist devices more often than postchange era patients. More patients were supported on temporary circulatory assist devices preoperatively in the postchange era than the prechange era (14 [24.1%] vs 0; *P* < .001). A total of 6 patients (10.3%) in the postchange era and 1 patient (2.0%) in the prechange era underwent heart-kidney transplant. All recipients in the prechange era were residents of Connecticut, while 3 recipients (5.2%) in the postchange era were from out of state. Survival at 180 days was not significantly different between the 2 eras (43 [87.8%] in the prechange era vs 52 [89.7%] in the postchange era).

Volume increase at 4 other regional centers during the comparable time period was variable compared with ours, with percentage changes in volume ranging between −10% and 68%, while our center’s volume increased by 374% (Figure).

## Discussion

Our study suggests that strategic and service structure changes led by new surgical leadership coinciding with change in the UNOS allocation system may have substantially increased the acceptance of donor heart offers and transplant volume while maintaining comparable unadjusted short-term outcomes. The volume increase at our center was associated with accepting hearts from older donors with more comorbidities that were refused by more centers, and offering opportunities to recipients with higher acuity (ie, receiving extracorporeal membrane oxygenation or an intra-aortic balloon pump requiring heart-kidney transplants). These observations may be applicable to other centers contemplating increasing the use of donor hearts.

More than 50% of the hearts offered for transplant are not transplanted for various reasons,^7^ one of which is a clinician’s perception, which is potentially modifiable, that the particular heart is not suitable,^8^ and the higher sequence number in the postchange era implies that the hearts would have gone unused had we not accepted them. Because outcomes of low-volume centers are susceptible to sporadic deaths, Medicare’s reimbursement requirement to maintain a certain survival rate^9^ may encourage smaller centers to take a more conservative approach to donor heart selection. The current system of performance measure and reimbursement may incentivize a conservative approach because there is little benefit to the programs and hospitals to take on higher-risk donors and recipients while the penalty of risking mortality from being aggressive is quite high, including being on probation and potentially losing the credential to perform transplants. Although our prechange era volume was low, we were able to adopt an aggressive stance toward donor heart selection, supported by the hospital funding for a dedicated procurement team and transplant coordinators.

This increase in the transplant volume was accompanied by a decrease in the use of durable left ventricular assist devices. Although we did not have any concerns for the outcomes associated with implantations of the device, we were able to offer more patients a direct path to transplant and, as an unintended consequence, the volume of left ventricular assist device implantation declined in the postchange era. The total number of patients who underwent surgical advanced heart failure therapy (left ventricular assist device implant or heart transplant) increased in the postchange era compared with the prechange era: there were, on average, 42 such patients per year in the prechange era whereas there were 69 patients per year in postchange era. The exact cause of this increase is difficult to isolate but likely involves publicity regarding the increase in the propensity for transplants at our center.

Although our center’s changes coincided with the 2018 update in the UNOS status definitions, our center’s disproportionate increase in transplant volume compared with other regional centers indicates that the association of the status definition change alone with the volume increase was small. Because national posttransplant survival may be worse under this allocation system,^6^ acuity of the recipients and outcomes must be carefully monitored. Increasing the heart transplant volume at each center may improve national outcomes by increasing the number of high-volume centers to achieve excellent outcomes.^10,11^

### Limitations

This study has some limitations. This is a pre-post cohort study and a causal relationship between the program change and patient characteristics or case volume was not ascertained. However, the program changes coincided with the expected change in donor heart characteristics and increase in the case volume, suggesting that the observed changes were indeed associated with the program change. Comparing the clinical profile of recipients was limited to item-level bivariate analysis without the use of composite risk profiles because the definition of waiting list status changed around the time of our program change. We elected not to use an existing heart transplant risk score because of the limited discriminatory performance of such a score.^12^ Survival was assessed only up to 180 days and the risk of late consequences, such as allograft vasculopathy, associated with our approach requires ongoing investigation.

## Conclusions

Depending on centers’ current philosophy toward heart transplant, a strategic multidisciplinary change in donor heart and recipient selections may increase the number of heart transplants while maintaining short-term outcomes comparable with more conservative patient selection. Combined with the new UNOS status definition, this change may augment the allocation of currently unused donor hearts.
